# Mitochondrion: Main organelle in orchestrating cancer escape from chemotherapy

**DOI:** 10.1002/cnr2.1942

**Published:** 2023-12-27

**Authors:** Samaneh Mostafavi, Nahid Eskandari

**Affiliations:** ^1^ Department of Immunology, Faculty of Medical Sciences Tarbiat Modares University Tehran Iran; ^2^ Department of Immunology, Faculty of Medicine Isfahan University of Medical Science Isfahan Iran

**Keywords:** drug resistance, mitochondria, neoplasm, oxidation–reduction

## Abstract

**Background:**

Chemoresistance is a challenging barrier to cancer therapy, and in this context, the role of mitochondria is significant. We put emphasis on key biological characteristics of mitochondria, contributing to tumor escape from various therapies, to find the “Achilles' Heel” of cancer cells for future drug design.

**Recent findings:**

The mitochondrion is a dynamic organelle, and its existence is important for tumor growth. Its metabolites also cooperate with cell signaling in tumor proliferation and drug resistance.

**Conclusion:**

Biological characteristics of this organelle, such as redox balance, DNA depletion, and metabolic reprogramming, provide flexibility to cancer cells to cope with therapy‐induced stress.

## 1 INTRODUCTION

Despite the huge efforts made to cure cancer, this disease is still the most important cause of death worldwide, with 10 million deaths in 2020.^1^ Tumor cells acquire resistance to therapy via several mechanisms, including transporter pumps, oncogenes (EGFR, PI3K/AKT, ERK, and NF‐κB), tumor suppressor genes, exosomes, DNA repair, autophagy, epithelial‐mesenchymal transition (EMT), cancer stemness, and mitochondrial alteration.^2^ Mitochondria are sites for common metabolic reactions, known as powerhouses of cells, and provide adenosine triphosphate (ATP) for tumor rapid growth and spread.^3^, ^4^ However, mitochondria are not only the powerhouse of cancer cells. In the metabolic reprogramming of cancer cells, there are at least five mechanisms by which mitochondria are involved in cancer initiation and the development of therapy resistance. First, alteration in subunits of the electron transport chain (ETC).^5^ Studies have demonstrated that mutations in mitochondrial genes encoding complex I components may induce reactive oxygen species (ROS)‐dependent oncogenic pathways.^6^ The importance of ETC‐mediated ROS generation in tumorigenesis and therapy resistance has been proven in breast cancer, pancreatic cancer, and oral cancer.^7^ ROS is the second mechanism by which mitochondria contribute to cancer initiation. Indeed, the tricarboxylic acid (TCA) cycle and the ETC are the main pathways for generating ROS and superoxide anions, which are byproducts of oxidative respiration. The increase in metabolic activity of cancer cells is directly correlated with an increase in ROS in the tumor microenvironment. Third, mitochondria are the main organelles for initiating and regulating cell death via apoptosis. Hence, mitochondrial proteins like VDAC, BCL‐2, and the Mcl‐1 family modulate apoptosis and play crucial roles both as oncogenic and oncosuppressive factors.^8^ Fourth, mitochondrial metabolic adaptation in cancer promotes metabolic flexibility to cope with cellular stress and maintain ATP production to overcome therapy. In this context, genes encoding TCA enzymes cause several mutations, resulting in the accumulation of TCA metabolites such as fumarate, succinate, aspartate, and D‐2‐hydroxyglutarate (2HG), thereby promoting pro‐carcinogenic effects.^9^ Moreover, oncogenic signaling pathways affect mitochondria. For instance, the PI3K‐PTEN‐AKT pathway shifts oxidative metabolism toward glycolysis. However, changes in the cellular redox status and ROS generation by mitochondria contribute to tumor proliferation and therapy resistance.^10^ Fifth, the telomerase reverse transcriptase (TERT) transition from the nucleus to the mitochondria plays a role in preserving mitochondrial DNA (mtDNA) from oxidative stress and apoptosis.^11^ Hence, mitochondria are key organelles in cancer progression and play pivotal roles in drug resistance, which is worth reviewing. Here, we discussed the key biological characteristics of mitochondria, which contribute to cancer drug resistance.

## 2 ROLES OF mtDNA AND CANCER DRUG RESISTANCE

The relationship between mtDNA and cancer is controversial. Studies demonstrated that an increase in mtDNA content is correlated with the advanced stages of esophageal squamous cell carcinoma and head and neck squamous cell carcinoma.^12^, ^13^ On the other hand, low mtDNA content has been identified to be associated with invasive forms of lung cancer, ovarian carcinoma, and breast cancer.^14^, ^15^, ^16^ Moreover, squamous cell carcinomas with lower mtDNA content were less responsive to cisplatin.^17^ Cancer cells mostly contain alterations and/or somatic mutations in the mtDNA, resulting in mitochondrial dysfunction in these cells. It has also been observed that mtDNA content is reduced in tumor‐initiating cells, which are thought to play a role in cancer recurrence after chemotherapy.^18^ Cancer cells often have mutations in mtDNA, but these mutations do not completely impair the mitochondria‐mediated energy metabolism and functionality.^19^ Rather than that, these mutations promote alteration of the biosynthetic and bioenergetic characteristics of the cancerous cell via mitochondria‐to‐nucleus signaling. This signaling pathway is activated by “dysfunctional” mitochondria that lead to mitophagy and alter the activity and/or transcription of cancer‐related genes and signaling pathway.^20^ According to a study by Wang et al.,^21^ mitochondrial dysfunction resulted in cisplatin resistance in human gastric cancer. However, regulation of defective mitochondria in colorectal cancer improved drug resistance by increasing ROS and trigging mitochondrial stress.^22^

Comparisons between mtDNA‐depleted hepatocarcinoma cells (Rho cells) and wild‐type cells demonstrated that due to the activation of NRF2‐mediated pathways in Rho cells, the expression of MDR1, MRP1, and MRP2 was increased, leading to a decrease in sensitivity to doxorubicin, SN‐38, and cisplatin.^23^, ^24^ Moreover, in a study on oral squamous cell carcinoma (OSCC), it was shown that cells with lower mtDNA content were less responsive to cisplatin. However, cisplatin‐resistant cells utilized a metabolic shift toward glycolysis, and based on the findings of this study, this metabolic shift probably contributed to mtDNA depletion.^17^ Furthermore, mtDNA, compared with nucleus DNA, is much more exposed to ROS‐derived damages due to the source of ROS generation. Studies on mitochondrial DNA‐depleted glioma cells (ρ°), treated with temozolomide, have shown that ρ° cells are resistant to therapy and also have lower levels of ROS compared with controls. In this study, chemoresistant glioma cells were also insensitive to the bulk of ROS generation and hydrogen peroxide challenges.^25^

**FIGURE 1:**
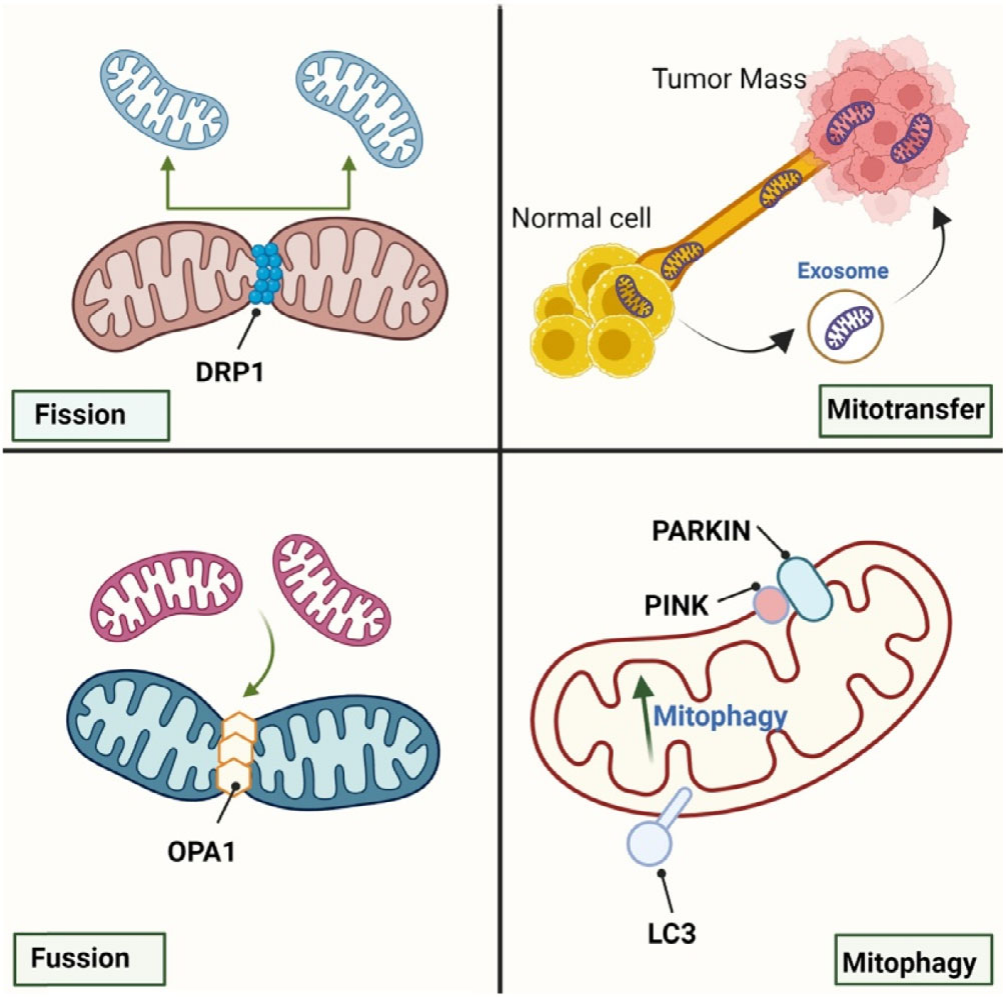
Mitochondrial dynamics in cancer Mitochondrial fission and fusion by altering and modulating mitochondrial biogenesis may affect therapy; however, normal cells, by transferring mitochondria to tumor mass, serve energy production and organelle regeneration in tumors. Mitophagy also disrupts chemotherapy by destroying damaged mitochondria.

MtDNA mutations provide tumor cells with a flexible and rewired metabolism to tolerate drug‐induced stress. Cells without this flexibility would die, and resistant cells with specific mtDNA mutations would endure treatment. These mutations determine the response to therapy. For instance, in ACR20 cells (cisplatin‐resistant cells derived from A549 lung cancer), findings suggest that intrinsic ROS levels were elevated by mitochondrial DNA mutations, which decreased the sensitivity to cisplatine (cis‐diamminedichloroplatinum(II) (CDDP)) via activation of NF‐κB signaling and induction of Inhibitor of apoptosis expression in these cells.^26^ Also, hormonal therapy‐resistant breast cancer cells derived from patients utilize the packing and transferring of mitochondrial DNA via stromal exosomes.^27^ Iintracellular mitochondrial transfer or mito‐transfer (incorporation of mitochondria or fragments of them and mitochondrial DNA), is beneficial for tumor growth even in cancers with fully functioning mitochondria, which is enhanced during chemotherapy (Figure 1).^28^ Therefore, targeting mtDNA can be one of the most effective strategies to overcome cancer drug resistance.

## 3 MITOCHONDRIAL REDOX HOMEOSTASIS IN CHEMORESISTANCE

**FIGURE 2:**
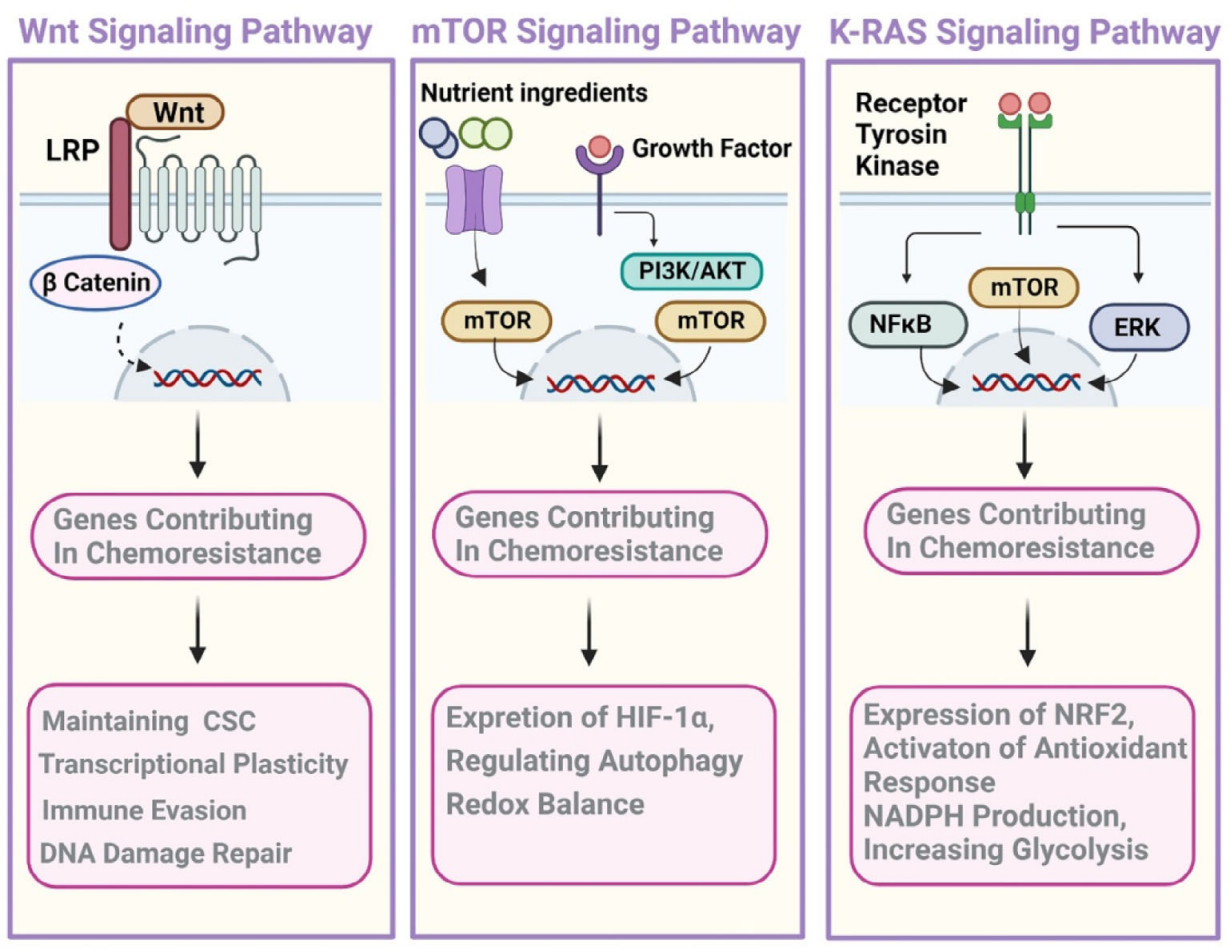
The main signaling pathways contribute to chemoresistance. Wnt, mTOR, and K‐RAS are known as critical signaling pathways. Genetic rewiring alters these pathways by enhancing and regulating genes like HIF1 and NRF2, which not only reprogram cancer metabolism but also enhance scavenging chemotherapy‐mediated reactive oxygen species to save DNA from damage and eventually apoptosis. CSC, cancer stem cell; NADPH, nicotinamide‐adenine dinucleotide phosphate.

Although ROS has several sources, including proteins within the plasma membrane, such as the growing family of NADPH oxidases and lipid metabolism within the peroxisomes, as well as the activity of various cytosolic enzymes such as cyclooxygenases, 90% of cellular ROS is produced by mitochondria.^29^ The mechanisms of ROS‐derived chemoresistance via ER stress‐mediated autophagy are to increase cellular proliferation, metastasis, and the generation of cancer stem cells (CSC).^30^ Generally, chemotherapy by increasing ROS and altering redox stability triggers apoptotic mechanisms in cancer cells; however, one of the mechanisms of malignant cells to overcome such therapies is overexpression of the antioxidant machinery enzymes, including superoxide dismutase (SOD), peroxiredoxins, catalase (CAT), and glutathione peroxidase (GPx).^31^ Moreover, the pentose phosphate pathway (PPP) improves the NADPH pool, and redox homeostasis counteracts oxidative stress, thus allowing cancer cells to develop resistance to cisplatin.^32^, ^33^ IIn this regard, the ratio between activated pyruvate kinase (PK), one of the enzymes of glycolysis, and the inactivated form of this enzyme determines whether glucose should be used for either oxidative phosphorylation (OXPHOS) to produce energy or for PPP to detoxify ROS.^34^ ROS of mitochondria via interacting with the permeability transition pore complex (PTPC) or pro‐apoptotic proteins (e.g., BAX and BAK) that control mitochondrial outer membrane permeability (MOMP) triggers mitochondrial‐derived apoptosis.^35^ Therefore, redox homeostasis seems to be inevitable for tumors, and one of the main organelles that contributes to this event is the mitochondrion. Activation of the PI3K/AKT pathway, K‐RAS, B‐RAF, and MYC signaling induce NRF2 nuclear translocation, leading to redox homeostasis alterations (Figure 2).^36^, ^37^

ROS has a dual role in cancer, either through induction of reprogramming in response to various environmental factors or via initiation of mitochondrial‐mediated apoptosis.^38^ OXPHOS of mitochondria is one of the undeniable sources of ROS, in which superoxide anion radical (O_2_˙^−^), hydrogen peroxide (H_2_O_2_), and the hydroxyl radical (OH^•^) are generated during ETC activity; hence, mitochondrial redox homeostasis mechanism, such as the above‐mentioned enzymes, strongly preserve mitochondria from ROS‐derived damages.^39^ Mitochondrial SOD (SOD2) converts superoxide anion radicals to H_2_O_2_, which is known as a second messenger, and based on the studies by Nerush et al, an increase in H_2_O_2_ occurred during cisplatin therapy of HeLa Kyoto cells, and led to apoptosis.^40^ But ROSs are not always harmful for cellular function, whereas some of them, including H_2_O_2_, and O_2_˙^−^,participate in cellular signaling, steroid synthesis, and gene expression to regulate cell defense mechanism.^41^

Cancer cells use mitochondrial‐derived strong antioxidant machinery, thereby suppressing ROS levels, among which sirtuin‐3(SIRT3) is considered a key molecule.^42^ SIRT3 affects mitochondrial metabolism, including TCA, urea cycle, amino acid metabolism, fatty acid oxidation, ETC/OXPHOS ROS detoxification, mitochondrial dynamics, and the mitochondrial unfolded protein response (UPR).^43^ Studies have exhibited that silencing of SIRT3 is along with decreasing mitochondrial function, and oxygen consumption, which suggests an appealing strategy to render colorectal cancer cells more sensitive to treatment.^44^ SIRT3 can also regulate mitochondrial SOD2, thereby scavenging ROS levels.^43^ Another study on colorectal cancer cells demonstrated that suppressing SIRT3 re‐sensitized malignant cells to anticancer drugs by inhibiting SOD2, increasing ROS levels, and down‐regulating mitochondria activity. Moreover, SIRT3 protein levels in specimens of colorectal cancer patients were directly correlated with survival.^45^ Studies conducted by Azwar et al.^46^, demonstrated that an increase in intracellular ROS may re‐sensitize colon cancer cells to 5FU by activating AMPK and blocking autophagic flux, which were as a result of ROS‐mediated impairment autophagolysosome accumulation. Furthermore, Gpx, which contributes to detoxification of H_2_O_2_ and activation of the PI3K/AKT pathway, also promotes the induction of chemoresistance to cisplatin in NSCLC cells. This enzyme also plays a role in the matrix of mitochondria, scavenging H_2_O_2._ ^47^ However, superoxide anion is the main free radical produced during OXPHOS, and mitochondria have competent mechanisms for free radical removal, hence enabling tumor cells to overcome chemotherapy via overexpression of antioxidant enzymes, which has been shown in a study on pancreatic cancer cells resistant to gemcitabine‐derived ROS via upregulation of SOD2 and CAT.^48^ Another study on the effect of antioxidants on chemoresistance demonstrated that the combination of cisplatin with polyphenols (like curcumin) leads to enhanced antioxidant effects and resistance to cisplatin.^49^ Following this line, mitochondrial inhibition via elevated ROS, which triggers mitochondrial‐dependent apoptosis, seems to be a promising therapeutic approach in chemoresistant cancer cells. A study by Zhang et al.^50^, on mitochondrial‐specific drugs demonstrated that in situ amplification of mitochondrial ROS by damaging mitochondria and cytochrome c (Cyto c) releases activated caspase3 and apoptosis of breast cancer cells, thereby enhancing chemotherapy.^50^ Therefore, mitochondria should be considered a critical and main responder organelle to drug resistance and apoptosis..

## 4 DYNAMIC OF MITOCHONDRIA IN CHEMO‐RESISTANT CANCER CELLS

Mitochondrial dynamics (including fission, fusion, and mitophagy) are biological events determining cellular behavior (e.g., metabolism, signaling, cell proliferation, and migration). The importance of the dynamics of mitochondria has been proven in many tumors like ovarian, lung, hepatocellular carcinoma, and breast cancer.^51^ Based on recent research, cancer cells, via alteration of the dynamic of mitochondria, not only follow their aim in cell survival, metastasis, metabolic reprogramming, and tumor growth, but also overcome chemotherapy.^52^

Mitochondrial fusion (predominantly by a protein known as OPA1) provided a hyperfused mitochondrial network, increasing ATP production and inhibiting autophagy. On the other hand, mito‐fission (predominantly by a protein known as dynamin‐related protein 1 (Drp1)) divides or segregates mitochondria into two separate mitochondrial organelles (Figure 1).^53^ Mitochondrial dynamics serve to maintain the pool of mitochondria within a cell and optimal OXPHOS activity by providing efficient transport and distribution of mitochondrial content. For instance, OXPHOS is facilitated in fused mitochondria via coupled ETC complexes. Fission and fusion adapt cells for both properly accessing nutrients and covering energy demand.^54^ Mitochondrial dynamic roles as a switch and allows the organelle to respond to circumstances appropriately. For instance, during cancer cell generation, mitochondrial fission inhibits cancer proliferation and metastasis through inhibiting PI3K/Akt/mTOR and Ras/Raf/MEK/ERK signaling pathways. Treatment with leflunomide, a potent activator of mitochondrial fusion proteins, overcame the inhibitory effects of fission on migration, signaling, and metastasis. A study on breast cancer cells revealed that increased expression of genes associated with mitochondrial fission correlated with improved survival in human breast cancer.^55^

A study on cisplatin‐resistant ovarian cancer cells provided evidence that the inhibition of Drp1 hindered mito‐fission and consequently re‐sensitized ovarian cancer cells to cisplatin.^56^ Another study on these cells also revealed that under hypoxia, mitochondrial efficacy decreased, and downregulation of Drp1 reversed the cisplatin resistance.^57^ Chemoresistant cancer cells have more integrated mitochondria, which may protect them from CytoC release and apoptosis.^58^, ^59^ Inhibition of mitochondrial fusion by silencing mitofusin (MFN1), an important protein involved in the dynamics of mitochondria, may contribute to re‐sensitizing GBM calls to cisplatin.^60^ Moreover, inhibition of mitophagy in breast cancer via induction of mitochondrial fission sensitized these cells to chemotherapy.^61^ Furthermore, phosphorylation and activation of Drp1 are correlated with brain tumor initiation, and it has been clarified that the disease progression is compromised by mitochondrial fragmentation.^62^ Moreover, down‐regulation of Drp1 via metformin increased the chemosensitivity of nasopharyngeal carcinoma (NPC) cells to cisplatin.^63^ Mitophagy provides homeostasis for the normal physiological activity of mitochondria.^64^ Cancer cells become resistant to drugs, targeting mitochondrial activity via scavenging damaged and dysfunctional mitochondria through mitophagy to mediate their own drug resistance.^65^ Hence, by targeting mitochondrial biogenesis through drugs like metformin, these cells become resistant to treatment.^66^ Moreover, Drp1 phosphorylation via ERK1/2 signaling activated autophagy in chemoresistance colorectal cancer cells, which resulted in the regrowth of surviving tumor cells.^67^

## 5 METABOLIC ADAPTATION OF MITOCHONDRIA IN CHEMO‐RESISTANCE CANCER CELLS

**FIGURE 3:**
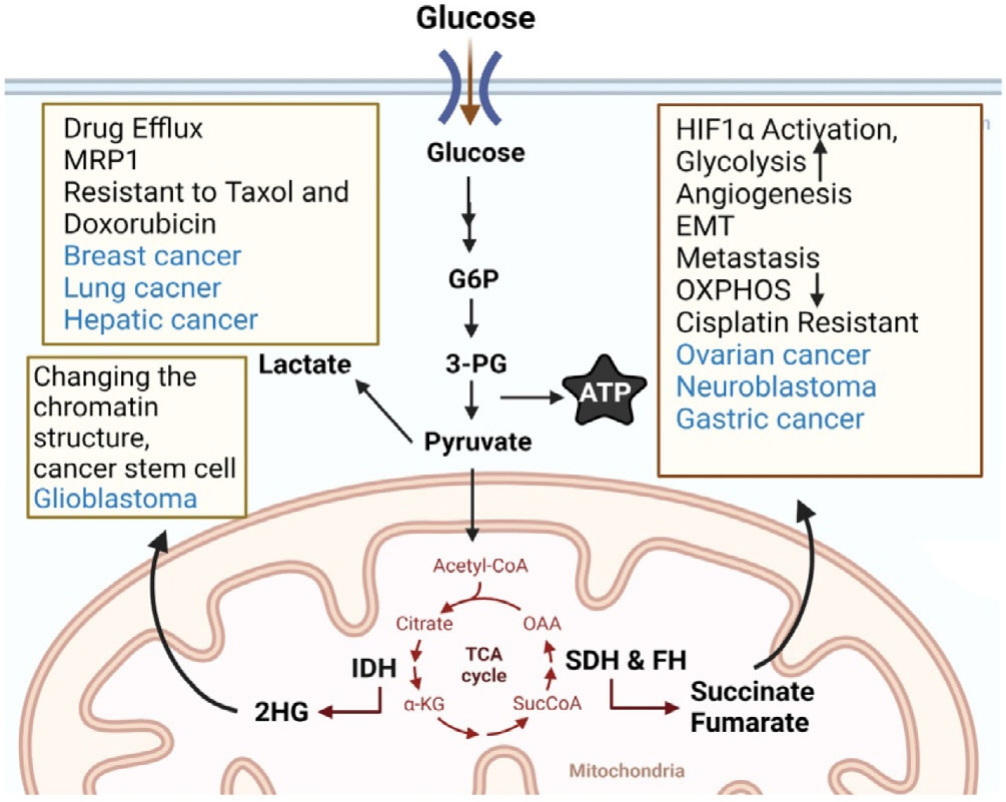
The main mechanisms of mitochondrial‐mediated oncometabolites in chemoresistance. 2‐hydroxyglutarate (2HG), fumarate, succinate, and lactate are known oncometabolites in cancer, affecting the efficacy of chemotherapy for several tumors. Cancer cells utilize these oncometabolites to rewire the metabolism and signaling pathways in response to therapies for tumor evade and chemoresistance. SDH, succinate dehydrogenase.

One of the main hallmarks of cancer is metabolic reprogramming, which refers to the high demand for glucose consumption. During metabolic reprogramming, cancer cells convert pyruvate into lactate, regardless of hypoxia or normoxia status, under the action of lactate dehydrogenase (Figure 3).^68^ Based on the “Warburg effect”, cancer cells are addicted to glycolysis, and the glucose consumption rate of these cells is very high; therefore, Warburg assumed that it is probable that mitochondrial OXPHOS is impaired in these cells.^69^, ^70^ It is not clear why cancer cells prefer the Warburg effect (aerobic glycolysis); however, a study on cancer metabolism showed that it is probably due to providing metabolites for the PPP.^71^ The importance of PPP for cancer cells refers to nicotinamide‐adenine dinucleotide phosphate (NADPH) production, which is needed for redox homeostasis and lipid metabolism, as well as nucleotide production, which is essential for cancer proliferation and DNA synthesis.^33^

Chemoresistant cancer cells may undergo metabolic reprogramming.^72^ A study by Roberta et al.^73^, on breast cancer cells treated with doxorubicin, cisplatin, and tamoxifen showed that chemotherapy shifted cancer metabolism toward fatty acid synthesis. Glycolysis and lactate production in chemotherapy‐treated cells are enhanced compared with control, which indicates that acidosis might increase in drug‐received tumors. Whereas, tamoxifen‐treated cancer cells did not show significant changes in lactate production. However, cisplatin and doxorubicin remarkably increased lactate and acidosis.^73^ Moreover, it has been demonstrated that treatment of tumors with antiangiogenic drugs by reducing tumor vascular density enhances hypoxia, which in turn induces a metabolic shift toward lipolysis.^74^ Lipid metabolism serves as an alternative metabolic pathway in stress conditions when glycolysis is hindered (such as exposure to MAPK inhibitors, which strongly decrease glucose uptake and glycolysis). Therefore, shifting to lipid metabolism adapts tumor cells to metabolic stress, which is induced by metabolically targeted therapies such as MAPK inhibitors.^75^

While mitochondrial turnover, mitophagy inhibition, and fully functional mitochondria resulted in cisplatin resistance in human ovarian cancer,^76^ targeting mitochondrial‐related metabolic pathways could be a promising therapeutic target for cancer therapy.^77^ In this context, some mitochondrial metabolic targeted therapies have been introduced, such as biguanides like metformin.^66^ The promising therapeutic effects of metformin have been approved in both experimental and clinical studies on several cancers, such as ovarian cancer, lung adenocarcinoma, and breast cancer.^78^, ^79^, ^80^ Metformin targets complex I of mitochondrial ETC, inducing metabolic stress and apoptosis in tumor cells.^81^ In addition, metformin rewires lipid metabolism through the inhibition of OXPHOS and activation of the TCA cycle. However, resistance to metformin is also probable since mitochondria endure dysfunctional ETC by up‐regulating alternative mitochondrial metabolic pathways such as an increase in the influx of glutamine into the TCA cycle.^66^

CSCs are dependent on OXPHOS for energy production compared with non‐stem cells; therefore, inhibition of mitochondrial activity and ATP pool may result in CSC apoptosis.^82^ In this regard, a study by Sancho et al.^83^ demonstrated that pancreatic CSC metabolism is highly dependent on mitochondria, and signaling transduction of C‐MYC is a key pathway in drug‐resistant cells. Therefore, inhibition of C‐MYC could be a promising way for re‐sensitizing resistant colons of pancreatic cancer to therapy.^83^

Recently, scientists have found a crucial relationship between mitochondrial calcium uniport (MCU) and aerobic glycolysis in cisplatin‐resistant ovarian cancer cells.^84^ MCU imports Ca^2+^ from cytosol to the mitochondria, preventing cellular apoptosis.^53^ Based on studies by Chakraborty et al.^84^, chemoresistant ovarian cancer cells are not only dependent on glycolysis, but also MCU overexpression in these cells contributed to inhibition of Ca^2+^ and apoptosis. Therefore, by decreasing MCU expression, they were able to to re‐sensitize cells to cisplatin. MCU is one of the crucial mitochondrial hallmarks of chemoresistance, indicating the potential role of MICU1 in reprogramming the glycolysis pathway toward aerobic glycolysisand also suggesting the main role of MICU1 in maintaining aerobic glycolysis (lactate production) in ovarian cancer cells.^84^ Therefore, MICU1 may contribute to mitochondrial metabolic reprogramming in chemoresistant ovarian cancer cells.

Mitochondria are known as key organelles in cell life and death because communicate with the cytosol and other organelles via several molecules and proteins.^85^ One of the most important is the voltage‐dependent anion channel (VDAC), which is involved in cell survival and apoptosis and also regulates cellular metabolism via metabolite flux. VDAC is in the mitochondrial outer membrane (MOM), and acts as an anchor for several proteins.^86^ For instance, hexokinase (HK), a key enzyme in cancer metabolism, is one of these proteins. HK by promoting glycolysis, is considered a key enzyme in cancer progression and drug resistance, and its overexpression in cancer cells helps with maintaining the Warburg effect.^87^ Moreover, HK has a dual role in metabolism and apoptosis.^88^ Binding of HK to VDAC inhibits mitochondrial‐mediated cell death.^89^ Based on studies, rituximab chemoresistance is mediated by HK activity, and downregulation of this enzyme may re‐sensitize rituximab‐resistant cell lines to chemotherapeutic agents.^84^, ^90^

One of the anti‐tumoral mechanisms of cisplatin is inhibiting VDAC1 channel activity and inducing apoptosis by impairment of metabolic flux between mitochondria and cytosol, leading to MOM permeabilization and Cyto c release.^91^ Therefore, it is reasonable to consider VDAC1 as the main molecule in mechanisms of resistance to cisplatin, which needs to be more investigated.

Mitochondrial metabolic adaptation conferred resistance to PI3K therapy in GBM, and suppressing mitochondrial bioenergetics improved the efficiency of PI3K therapy.^92^ In cisplatin‐resistant ovarian cancer cells, a metabolic shift toward OXPHOS has been reported.^93^ Therefore, mitochondria play a key role in cisplatin‐resistant cancer cells. Over expression of pyruvate dehydrogenase kinase in mitochondria was observed in chemoresistant tumors, in which this enzyme was responsible for metabolic reprogramming toward glycolysis.^94^ Hence, tumors become resistant to therapy via different metabolic pathways, enabling them to provide energy via alternative ways for their rapid growth. Overcoming drug resistance isn't a global pattern, but it depends on the tumor subtypes and should be studied well.

Studies by Prasad et al.^95^, demonstrated that in the core of solid tumors there is glutamine starvation, resulting in metabolic differentiation in cancer cells toward enhanced glycolysis and an increase in proteins involved in mitochondrial dynamics such as DRP1. In this study, it has also been shown that up‐regulation of DRP1 in glutamine starved tumors results in the mitochondrial fission, contributing to chemoresistance in tumors like cervical cancer, lung cancer, and ovarian cancer.^95^ Metabolism of glutamine via mitochondria plays a pivotal role in chemoresistant cells, which should be considered a challenge for re‐sensitizing cancer cells. Administration of inhibitors specific to different factors of glutamine metabolism along with chemotherapy may overcome chemoresistance.^96^ In vitro studies demonstrated that mitochondrial OXPHOS directly affect the cytotoxicity of cisplatin in lung cancer cells. In fact, cells with enhanced OXPHOS are more sensitive to cisplatin.^97^

Studies on colorectal cancer and chondrosarcoma revealed that enhanced aerobic glycolysis contributed to chemoresistance, and targeting glycolysis improved cisplatin‐induced apoptosis in ovarian cancer.^98^, ^99^, ^100^ Doxorubicin‐resistant MCF‐7 cells, however, became re‐sensitized to doxorubicin via activation of AMPK pathway, which is directly correlated with glycolysis.^101^ Studies by Zhang et al.^102^, demonstrated that hexokinase 2 confers enhanced glycolysis in cisplatin‐resistant ovarian cancer cells, which promotes autophagy in these cells. The main organelle in lipid metabolism, mitochondria, may alter and adapt lipid metabolism to endure chemotherapy‐derived cellular stress; therefore, rewiring lipid metabolism could be a protective response to stress.^75^ Lipid metabolic reprogramming consequently increases in fatty acid oxidase, to provoke glutathione production, increases in NADPH levels for redox homeostasis, and overcomes therapy‐derived oxidative stress.^103^ Furthermore, mitochondrial activities in fatty acid metabolism, including fatty acid β‐oxidation and TCA cycle, are involved in the chemoresistance of GBM cells to TMZ, about which studies indicated that prostaglandin E2 plays a pivotal role in regulating mitochondrial metabolic genes expression.^104^ Therefore, it seems that modulation of arachidonate metabolism may effectively re‐sensitize GBM cells to TMZ.

Evidences have shown that dysregulated lipid metabolism could be the reason for chemoresistance in colorectal cancer treated with 5‐fluorouracil and oxaliplatin in vitro and in vivo.^105^ Also, lipid metabolism is a key pathway in lung cancer resistance to EGFR tyrosine kinase inhibitors (EGFR‐TKIs).^106^ Mitochondrial lipids, such as cardiolipin, ceramide, and sphingosine‐1‐phosphate, activate mitophagy to overcome chemoresistance‐mediated mitochondrial injury.^107^ A significant decrease in ceramide was significantly correlated with the resistance of colorectal cancer cells to 5‐FU.^108^ In a study by Bermúdez et al.^109^, it has been shown that cisplatin‐resistant lung cancer cells increased in mitochondrial function and mass, as well as reduced glycolysis. However, inhibiting OXPHOS via metformin may overcome resistance in these cells. Studies on patients suffering from colorectal cancer revealed that an increase in OXPHOS is dependent on PGC1α, a particular molecule in mitochondrial metabolism and biogenesis.^110^ Elevated PGC1α could increase ROS production.^111^

Enzymes like adenylate kinase 4 (AK4), which are involve in cellular energy homeostasis, and hypoxic response modulation play a pivotal role in chemoresistance. This enzyme, by activating AMPK and HIF‐1α, promotes inflammation.^112^ Down‐regulation of AK4 re‐sensitized hela cells to the anti‐cancer drug, CDDP, which is along with a decrease in proliferation and tumor size by affecting mitochondrial TCA activity.^113^ Among cancer cells, serous ovarian cancer indicated high metabolic heterogeneity, in which the dominant metabolism of one group was glycolysis while in other cells it was OXPHOS. In addition, mitochondrial respiration, which generates oxidative stress in ovarian cancer, promotes sensitivity to conventional chemotherapeutic drugs.^114^

Mitochondrial ATP homeostasis is related to drug resistance.^115^ During normal conditions, OXPHOS produces ATP in mitochondria and normally hinders NOX4‐derived ROS, whereas during metabolic reprogramming of cancer cells and switching to glycolysis, ATP is generated in the cytosol and mitochondrial ATP homeostasis is disrupted, which leads to NOX4 activation. As shown in a study on renal cell carcinoma (RCC), NOX4 is an ATP‐binding motif within the NADPH‐oxidized isoform and located in the inner membrane of mitochondria. NOX4 is negatively regulated by directly binding to ATP, and down‐regulation of which is correlated with improving the drug efficacy in RCC.^116^ However, inhibiting mitochondrial complex I by down‐regulating oxygen consumption rate, and OXPHOS may re‐sensitize melanoma cells to chemotherapy with vemurafenib.^117^ Likewise, previous studies on metabolic reprogramming of cancer cells demonstrated that tumor cells prefer to switch to glycolysis (HIF‐1α is a key factor in metabolic rewiring toward glycolysis) to avoid mitochondrial respiration and ROS production, which is a strategy to directly reduce DNA damage and apoptosis.^77^, ^109^, ^118^ However, studies on lung adenocarcinoma cells have shown that cancer cells could become resistant to paclitaxel via defective mitochondrial respiratory function and down‐regulating citrate levels. Glycolysis in these resistant cells is a main metabolic pathway for ATP production; therefore, reducing the glycolysis (through activation of TCA and citrate production) could be a promising way for re‐sensitizing lung cancer.^119^, ^120^ A study on A594 lung cancer cell line showed that an increase in citrate down regulates tumor proliferation via several ways, like inhibition of glycolysis, the TCA cycle, and the IGF‐1R/AKT axis. In this study, citrate therapy improved the cisplatin chemotherapy outcome.^121^ However, a study on gastric cancer cell lines provided evidence that citrates not only re‐sensitized cancer cells to cisplatin, but also activated mitochondrial‐dependent apoptosis.^122^ Inhibition of mitochondria via metformin (as a mitochondrial complex I inhibitor) may induce chemosensitivity, as shown in ovarian cancer resistant to methotrexate and cisplatin.^123^ Metabolic reprogramming of cisplatin‐resistant lung cancer cells toward OXPHOS increased ROS generation. Therefore, cisplatin‐resistant lung cancer cells are dependent on redox balance (to counteract the high ROS levels) and glutamine metabolism for redox homeostasis. Treatment of cisplatin‐resistant cancer cells with riluzole, a medication used to treat amyotrophic lateral sclerosis and other motor neuron diseases, blocks glutamate flux, which may induce metabolic reprogramming in cisplatin‐resistant lung cancer cells in vitro and in vivo, resulting in apoptosis.^124^ Cisplatin‐resistant cancer cells have shown different metabolic rewiring. For instance, ovarian cancer cells are dependent on glycolysis; on the other hand, lung cancer cells increase in OXPHOS.^125^ Therefore, in studies on re‐sensitizing to chemotherapy, the tumor subtypes together with the types of chemotherapy regime are crucial and determining factors. Moreover, studies on molecular metabolism in resistant cells could be a promising therapeutic approach to overcome drug resistance (Table 1).

**TABLE 1:** Clinical trials on drugs, targeting mitochondria.

Tumor	Intervention/treatment	Effects on cancer	Identifier
Bladder cancer	Mitomycin C	Mitochondrial metabolic reprogramming toward OXPHOS, cytoplasmic release of mitochondrial DNA, inflammation Mitochondrial dysfunction, and decrease in complex I were seen in resistance cells	NCT04256122 NCT04256616
Colorectal cancer	CPI‐613	Targeting mitochondrial ATP production, improved the efficacy of 5‐fluorouracil	NCT02232152
Pediatric diffuse midline gliomas	Imipridone ONC201	Impaired tumor cell metabolism, mitochondrial damage, ROS production, and apoptosis	NCT04732065
Breast cancer, endometrial cancer	Small‐Molecule ONC201	Blocking mitochondrial activity	NCT03394027

The pivotal role of oncometabolites (succinate, fumarate, and 2‐hydroxyglutarate) in cancer progression and chemoresistance is undeniable (Figure 3). Indeed, succinate dehydrogenase (SDH) inhibition resulted in the accumulation of succinate, which induced pseudo‐hypoxic response by HIF1α activation. As a result, HIF1α downregulates OXPHOS and triggers glycolysis, GLUT1 expression, EMT, and angiogenesis for tumor metastasis in breast cancer.^126^ SDH is considered a crucial factor in cisplatin‐resistant ovarian^127^ and neuroblastoma cancer cells.^128^ In addition, cancer cells were indicated to have mutations in the fumarate hydratase (FH) gene, resulting in deficient FH and the accumulation of the fumarate. FH deficient cells displayed increased mitotic activity and resistance to ionizing radiations, as well as insensitivity to cisplatin.^129^, ^130^ Moreover, increased lipid metabolism influences 2‐hydroxyglutarate production from glucose in cisplatin‐resistant ovarian cancer cells.^131^ Moreover, an increase in 2‐hydroxyglutarate in GBM resulted in resistance to histone deacetylase inhibitors, maintaining CSC features and the self‐renewal properties of cancer cells.^132^ However, targeting lactate and lactate dehydrogenase provided promising results in re‐sensitizing breast cancer and hepatocellular carcinoma resistant to taxol and doxorubicin, respectively.^133^, ^134^ Lactate also enhances the expression of multidrug resistance‐associated protein 1 (MRP1), thereby increasing drug efflux and chemoresistance in lung cancer.^135^ Thus, regulating cancer metabolism to overcome drug resistance is a pivotal aspect of cancer therapy.

## 6 MITOCHONDRIAL STRESS ADAPTATION CONTRIBUTES TO DRUG RESISTANCE

As emphasized above, mitochondria are well orchestrated in response to cellular stresses, including cancer therapies. Mechanisms such as regulation of OXPHOS maintenance, ROS balance for signaling, modulation of apoptosis, and Ca^2+^ shuttle are necessary to overcome the therapy, which is carried out by mitochondria. Moreover, stress‐adaptive processes like mitochondrial dynamics and biogenesis, as well as interplay with other organelles, provide mitochondrial plasticity, enabling cancer cells to survive under stress conditions.^136^ In this context, the mitochondrial‐associated endoplasmic reticulum (ER) membrane (MAM) contribute to Ca^2+^ absorption and mitochondrial mediated drug resistance. For instance, restricting the transport of Ca^2+^ to the mitochondria in HeLa cells by MCU has been shown to induce resistance to therapy.^137^ Moreover, the quality control system of mitochondrial protein, which is in association with nucleus feedback, is controlled by several heat shock proteins and molecules. This includes the unfolded protein stress response (UPR^mt^), proteolytic stress response, and heat shock stress response. Alteration and reprogramming of these mechanisms in cancer cells and in response to therapy result in resistance to several treatments.^138^ More investigations are needed to clarify the specific strategies of mitochondria in controlling stress responses. These investigations may demonstrate mechanisms by which mitochondrial‐mediated drug resistance can be controlled.

## 7 TARGETING MITOCHONDRIA TO OVERCOME CANCER THERAPY

Targeting mitochondrial ETC is closely related to cancer cell apoptosis due to disrupting the mitochondrial redox balance. In this regard, a study on mitochondrial complex I demonstrated that inhibiting this complex inevitably triggered re‐sensitization to cisplatin, DOX, and 5‐FU.^139^ Moreover, metformin, as a new anticancer drug with potential efficacy in restricting drug resistance by inhibiting complex I, has been shown to contribute to increasing the sensitivity of lymphoma cells and lung cancer cells to therapy.^78^, ^140^ Moreover, recent studies have demonstrated that an increase in ROS contributes to drug resistance. For instance, resistance to gefitnib is associated with ROS generation and mitochondrial dysfunction in lung cancer cells.^141^ However, conventional therapies such as 5FU and cisplatin have been designed to increase ROS levels within the cells to initiate apoptosis.^142^, ^143^ Therefore, more investigations should be performed to explain the exact relationship between basal mitochondrial ROS range and drug resistance and to provide strategies for selective targeting of ROS.

The TCA cycle, which is an important pathway in generating ATP, is under investigation to explain the effects of its inhibition on drug resistance. One of the most prominent of these inhibitors is CPI‐613, which has been proven to have critical anti‐cancer activity in pancreatic cancer and AML. Moreover, CPI613 could sensitize cancer cells to cytarabine and mitoxantrone.^144^ The metabolism of glutamine is closely related to drug resistance. Therefore, a variety of glutaminase (GLS) inhibitors are under investigation, among which BPTES is one of the promising GLS inhibitors that efficiently increased the sensitivity of pancreatic cancer cells to a couple of therapies, and in renal cell carcinoma, another GLS inhibitor, known as CB‐839, is considered a potential drug.^145^ Moreover, targeting Ca^2+^ homeostasis in mitochondria may raise Ca^2+^ levels, resulting in re‐sensitizing to apoptosis. For instance, in ovarian cancer, it has been proven that Bcl‐2, through Ca^2+^ homeostasis, attenuates cisplatin cytotoxic effects, and targeting Bcl‐2 may result in overcoming therapy resistance in ovarian cancer.^146^ Therefore, targeting mitochondrial‐mediated mechanisms in drug resistance has garnered considerable interest recently. However, examinations, including preclinical and clinical, are needed to solidify previous results and determine if these approaches are effective in their actual clinical application. We summarized some of the clinical trials targeting mitochondria in Table 1.

## 8 CONCLUSION

Mitochondrial‐mediated biological aspects are key factors contributing to tumor progression and survival. Due to the main effects of mitochondria in drug resistance, targeting mitochondria is a promising therapeutic approach to re‐sensitize tumors. Mitochondrial‐mediated biological mechanisms, including mtDNA gene adaptation, mitochondrial dynamics, and metabolic reprogramming, are together mitochondrial pathways contributing to cancer cell adaptation in response to therapy. For instance, it has been indicated that there is a crucial association between the high rate of OXPHOS in cancer cells and drug resistance. Also, mitochondria, by reprogramming their number, structure, and location, increase the flexibility of cancer cells in response to stress conditions.^147^ TThis review paper aimed to introduce and centralize mitochondria as a main organelle, contributing to and orchestrating cancer drug resistance. We demonstrated that there are several unique molecular changes between 'oncogenic mitochondria' and normal healthy mitochondria, thereby carrying and transferring malignant information. These molecular features are particularly resulting from mtDNA and environmental reprogramming (such as PH, pO_2_, nutrients availability, and subject to treatment). In this context, several studies attempted to clarify the mechanism behind cancer drug resistance and aimed to target mitochondria. Therefore, it is worth noting that further examinations are needed to approve the efficacy and safety of mitochondrial‐targeted therapies.

## AUTHOR CONTRIBUTIONS

**Samaneh Mostafavi:** Conceptualization (lead); writing – original draft (lead); writing – review and editing (lead). **Nahid Eskandari:** Writing – review and editing (supporting).

## CONFLICT OF INTEREST STATEMENT

The authors have stated explicitly that there are no conflicts of interest in connection with this article.

## ETHICS STATEMENT

Not applicable.

## Data Availability

Data sharing is not applicable to this article as no new data were created or analyzed in this study.
